# Rationale and Design of RNAFH Study: Effect of Rosuvastatin (10 mg/d) on Nonalcoholic Fatty Liver in Metabolic Syndrome Patients without Overt Diabetes Evaluated by ^1^H-Magnetic Resonance Spectroscopy

**DOI:** 10.1155/2016/8454751

**Published:** 2016-10-19

**Authors:** Fan Ping, Xuan Wang, Jing Yang, Mei-cen Zhou, Wei Li, Ling-ling Xu, Yu-xiu Li

**Affiliations:** ^1^Department of Endocrinology, Key Laboratory of Endocrinology, Peking Union Medical College Hospital, Peking Union Medical College, Chinese Academy of Medical Sciences, Ministry of Health, Beijing, China; ^2^Department of Radiology, Peking Union Medical College Hospital, Peking Union Medical College, Chinese Academy of Medical Sciences, Beijing, China

## Abstract

*Objective*. The RNAFH study (effect of rosuvastatin on nonalcoholic fatty liver disease in metabolic syndrome patients without overt diabetes evaluated by ^1^H-MRS) is a prospective randomized, single-center, open-label trail designed to assess the effect of rosuvastatin on the intrahepatocellular lipid (IHCL) level of nonalcoholic fatty liver disease (NAFLD).* Methods*. 40 NAFLD patients meeting inclusion and exclusion criteria with metabolic syndrome (MS) but without overt diabetes mellitus will be included. Patients will be randomized to 52-week treatment with either rosuvastatin (10 mg/d) or blank control. The primary end point is IHCL evaluated by ^1^H-MRS, which was considered to be the most accurate noninvasive method for the evaluation of NAFLD. Secondary end points include homeostasis model assessment of insulin resistance (HOMA-IR) index on behalf of insulin resistance level and lipid parameters. Safety indicators will be monitored such as liver function, renal function, muscle stability, and glucose metabolism. The aims of the present study are noteworthy in respect that (1) IHCL is a quantitative indicator for evaluating the degree of fatty liver disease and ^1^H-MRS is a noninvasive technique to provide this specific index precisely, (2) meanwhile the HOMA-IR index and lipid parameters will be monitored, and (3) the safety of rosuvastatin treatment for 52 weeks will be evaluated including glucose metabolism, muscle stability, liver function, and renal function.

## 1. Introduction

Nonalcoholic fatty liver disease (NAFLD) refers to the presence of hepatic steatosis without other causes (e.g., heavy alcohol consumption) that can be found for secondary hepatic fat accumulation. NAFLD may progress to cirrhosis and is likely one of the most important causes of cryptogenic cirrhosis [1]. NAFLD is seen worldwide with the prevalence increasing over time and the major risk factors include central obesity, dyslipidemia, and impaired glucose metabolism which are also the main components of metabolic syndrome (MS) [2, 3]. The close relationship between NAFLD and MS has been demonstrated in a previous study, which showed that MS was associated with an increased risk of severe nonalcoholic fatty liver fibrosis (OR 3.5, 95% CI 1.1–11.2) [4]. On the other hand, NAFLD was an independent risk factor in the development of type 2 diabetes, especially in normal weight individuals with prediabetes. In addition, NAFLD has an important role in the pathogenesis of cardiometabolic diseases. It has also been demonstrated to be the strongest determinant of increased carotid intima-media thickness, which can stratify cardiovascular risk [5]. So far, there is no widely accepted pharmacologic treatment for NAFLD. The traditional treatment includes pioglitazone, a type of insulin-sensitizing agent, which could improve liver biochemical and histological parameters in patients with nonalcoholic steatohepatitis (NASH). However, its use was associated with adverse events, such as weight gain and swollen legs [6–10]. RCT studies with new agents have been conducted, but more extensive trials with larger sample size are needed. For example, liraglutide is a type of glucagon-like peptide 1 receptor agonist, which could resolve definite NAFLD and prevent the progression of fibrosis [11]. Elafibranor is an agonist of the peroxisome proliferator-activated receptor-*α* and receptor-*δ*, but its effect on NASH patients was uncertain, depending on the model of analysis [12]. The bile acid derivative 6-ethylchenodeoxycholic acid (obeticholic acid) is a potent activator of the farnesoid X nuclear receptor, which was shown to improve the liver histology of NASH patients proven by biopsy, but its long-term benefits and safety need further clarification [13].

A few RCT [14–17] studies have shown that the statins, the inhibitor of 3-hydroxy-3-methylglutaryl-coenzyme A (HMG-CoA) reductase, can improve the structure or liver function indices in patients with NAFLD and NASH. And also, they are safe to use in patients with chronic liver diseases and compensated cirrhosis even at high dose [16–18]. A recent observational study showed that statin use was protective from liver steatosis, steatohepatitis, and fibrosis stage in patients negative for the I148M variant [19]. Conversely, negative findings were also obtained in RCT19 where patients with biopsy-proven NASH were randomized to simvastatin versus placebo. However, these data were not convincing enough because (1) the sample size was too small [14, 16, 20] or the study duration was too short [16], (2) the other oral drugs like Vitamin C and Vitamin D were simultaneously taken, which might become the confounding factors [14], (3) diagnostic method was not accurate enough [14, 15], and (4) the primary end point was not the change of liver fat content or resolution of NAFLD or NASH [16, 17]. Further RCTs of adequate size and duration are required in the future.

## 2. Methods

### 2.1. Study Design

The RNAFH study is a prospective randomized, single-center, open-label study. This investigator-driven study is supported by AstraZeneca. In order to rule out confounding factors and meet the ethical standards to the maximum extent, this study excluded patients with overt diabetes. Interestingly, a very similar study was undertaken, which demonstrated that an inhibitor of 11 beta hydroxysteroid dehydrogenase type 1 (HSD1), namely, RO5093151, could decrease liver fat content in NAFLD patients. In that study NAFLD was also defined by ^1^H-MRS without liver biopsies, and diabetes patients were excluded because various diabetes treatments made it difficult to match the two groups [21].

In addition, ^1^H-MRS was currently confirmed to be the most sensitive, specific, noninvasive, and no-radiation method for the diagnosis of NAFLD, compared with CT, MRI, or US; the most attractive advantage was quantitative measurement of intrahepatocellular lipid (IHCL) [22]. Also, the reproducibility of this procedure was validated by duplicate hepatic triglyceride content measurements highly correlated and the coefficient of variation below 10%. The 95th percentile of IHCL in normal subjects was 5.56% [23]. It is known that NAFLD patients receive very different prognoses, depending on the degree of severity. Patients with mild disease are usually not eligible for pharmacologic therapy; instead the disease should be managed through dietary and lifestyle changes [12] Histological grade 1 of NAFLD (5%–33% macroscopic liver fat) corresponds to IHCL value of 11% [24]. According to this, only the patients with IHCL value above 10% are considered to have moderate to severe degree of liver steatosis. Therefore, the RNAFH study was focused on the effect of rosuvastatin on this group of patients with moderate to severe degree of NAFLD.

At maximal prescribed doses, rosuvastatin has the highest capacity for LDL-cholesterol (LDL-C) reduction and triglycerides (TG) lowering among the statins. Moreover, rosuvastatin has better absorption rate and bioavailability, and it is hydrophilic and hence associated with less adverse events [25]. In addition, rosuvastatin has similar or even less risks of hepatic dysfunction compared with other statins. So rosuvastatin is the most potent and relatively safe agent to choose in this study [25–27]. According to a recent meta-analysis, the risks of diabetes with satins versus placebo (OR 1.11) and intensive versus moderate intensity statin therapy (OR 1.12) were both within the acceptable range [28].

Approval of the protocols and informed consent forms was obtained from the institutional ethics committee of the Peking Union Medical College Hospital of Chinese Academy of Medical Sciences. This study has been registered in Clinical Trails.gov (ChiCTR-IPR-15007014).

### 2.2. Study End Points

This study is to explore the effect of rosuvastatin 10 mg/d on intrahepatocellular lipid (IHCL) in nonalcoholic fatty liver disease (NAFLD) patients with metabolic syndrome (MS) but without overt diabetes. The primary end point is the change of IHCL evaluated by ^1^H-MRS. Secondary end points are (1) lipid parameters including CHO (cholesterol), LDL-C (low density lipoprotein cholesterol), HDL-C (high density lipoprotein cholesterol), TG (triglyceride), and FFA (free fatty acid) and (2) HOMA-IR index calculated by fasting blood glucose (FBG) multiplied by fasting insulin (FINS) divided by 22.5. The safety indices are including ALT (alanine aminotransferase), AST (aspartate transaminase), CK (creatine kinase), TBIL (total bilirubin), FBG (fasting blood glucose), FINS (fasting serum insulin level), HbA1c (hemoglobin A1c), Scr (serum creatinine), UACR (urine albumin-creatinine ratio), and eGFR (estimated glomerular filtration rate).

### 2.3. Inclusion and Exclusion Criteria

40 NAFLD patients from the outpatient department of Peking Union Medical College Hospital meeting the criteria between January 2016 and January 2017 will be enrolled. All patients assessed for eligibility will be registered. Inclusion and exclusion criteria are shown as follows:

Inclusion criteria
  - Provision of informed consent prior to any study specific procedures
  - Men and female adults aged 18–70 years who agree to use contraceptive methods to prevent pregnancy while enrolled in study
  - Patients fulfilling the diagnostic criteria of MS of IDF in 2006 [3] (central obesity is an essential element (increased waist circumference which is ≥90 cm in Chinese men or ≥80 cm in women) plus any two of the following: TG > 1.7 mmol/L; HDL < 1.03 mmol/L in men or <1.29 mmol/L in women; SBP > 130 mmHg, DBP > 85 mmHG, or treatment for hypertension; FBG > 5.6 mmol/L)
  - The diagnosis of NAFLD screened by abdominal ultrasound and confirmed by ^1^H-MRS with IHCL > 10%
  - No overt diabetes history and FBG < 7.0 mmol/L plus HbA1C < 6.5%
  - Statins not used within past 3 months or currently taking fibrates
  - The patients without history of arteriosclerotic cardiovascular disease (ASCVD)
Exclusion criteria
  - Women planning to get pregnant within 1 year or being pregnant
  - TSH (thyroid stimulating hormone) > 10 uU/mL
  - Alcohol consumption per week >14 U (140 g) in women or >21 U (210 g) in men.
  - Positive results for any of HBsAg, HCV-Ab, and HIV-Ab or any other chronic or acute liver diseases
  - ALT or AST > 2 ULN (upper limits of normal) or TBIL > 2 ULN or CK > 2 ULN
  - eGFR (EPI) <30 mL/min
  - LDL-C < 70 mg/dL or LDL-C > 190 mg/dL
  - TG > 5.6 mmol/L
  - Allergy history of statins
  - systemic or inhalative steroid use within one year
  - Patients with active or chronic myopathy.

### 2.4. Study Procedure

**Figure 1:**
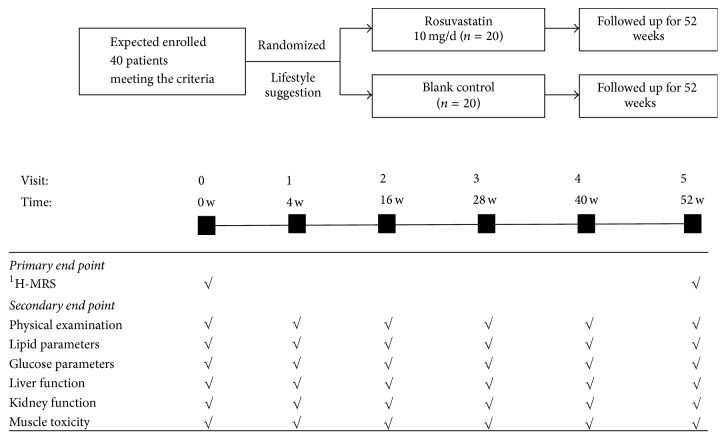
Flowchart showing the timetable of the RNAFH study. The total follow-up duration is 52 weeks. ^1^H-MRS will be performed at baseline and 52th week. Physical examination, lipid parameters, HOMA-IR, and safety indices including liver function, CK, renal function, and HbA1c will be measured at baseline, 4th, 16th, 28th, and 40th week, and 52nd week.

**Figure 2:**
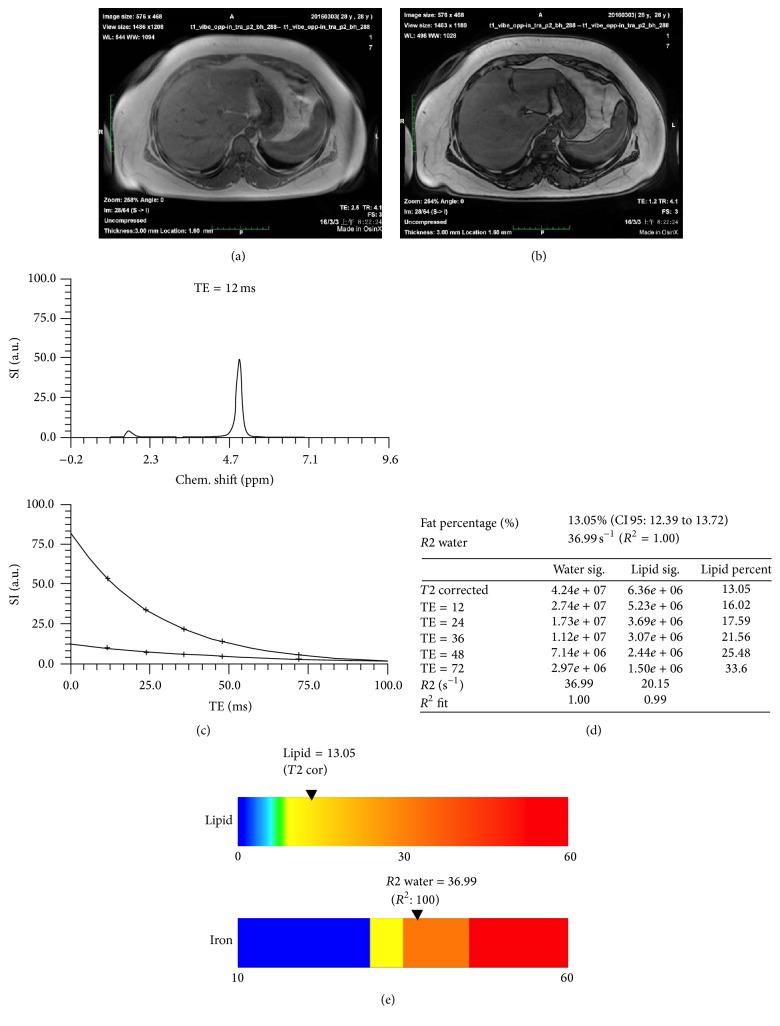
Axial breath-hold dual-echo T1-weighted imaging (in phase (a) and opposed phase (b)) of a subject enrolled with intrahepatocellular lipid (IHCL) of 13.05% (95% CI 12.39~13.72). The signal intensity loss of hepatic parenchyma on opposed-phase axial T1WI (b) image in comparison with in-phase image (a) indicates liver steatosis. The ^1^H-MRS voxel is placed on right lobe of liver. (c, d, e) are output images of MRS results, including (c) spectral peaks at TE 12 ms and T2 curve-fit of lipid and water, (d) a table of IHCL values calculated from MRS results, and (e) the color bars depicting lipid fraction and *R*2 water (iron) estimates.

The study flow is shown in Figure 1. Patients will be randomly assigned to receive rosuvastatin 10 mg/day (*n* = 20) or blank control (*n* = 20). All patients will be given lifestyle improvement suggestions at baseline. ^1^H-MRS will be conducted at the baseline and the 52nd week; the images of ^1^H-MRS are shown in Figure 2. Physical examination and ALT, AST, TBIL, eGFR (EPI), CK, Cr, ACR (urine), TSH, FBG, FINS, LDL-C, HDL-C, TC, TG, and FFA will be measured at the baseline, 4th, 16th, 28th, and 40th week, and 52nd week. Lifestyle improvement suggestions include reducing oil consumption, weight loss, increasing aerobic exercise, smoking cessation, and alcohol reduction. The lifestyle intervention should be recorded at each visit, including food frequency questionnaires and amount and intensity of exercise. The change of IHCL will be calculated by Siemens SKYIA 3.0 T using HISTO-MRS placed on the same region of right lobe of liver at baseline and at end point. Figure 2 showed the ^1^H-MRS images of one patient enrolled in this study with IHCL > 10%.

## 3. Determination of Sample Size and Statistical Analyses

The primary end point is the change of IHCL and there is no similar study for reference. Cowin et al. [29] conducted a study using ^1^H-MRS to evaluate IHCL change after a six-month weight loss program in overweight subjects. The IHCL by ^1^H-MRS before and after weight loss program were 15.2 ± 5.64% and 7.92 ± 6.18%, respectively. The change of IHCL was 7.28 ± 3.95%. We hypothesised that the effect of rosuvastatin (10 mg/d) on IHCL will be comparable to the effect of weight-loss. According to the study described above, we take 7.28 as the difference of mean of the change between two groups and 3.95 as the standard deviation. As two-side *α* = 0.05, *β* = 0.10, and actual power is 0.9, the sample size needed for each group is 7. Based on this assumption, the number of patients needed to be enrolled is 14, and considering the dropout rate of 20%, at least 20 patients with a total enrollment of 40 are needed.

IHCL change from baseline to 52 weeks after treatment will be compared between the control group and the rosuvastatin group using the two-sample *t*-test. Changes from baseline for the following parameters will be compared between the control group and the rosuvastatin group at 4 weeks, 28 weeks, and 52 weeks including LDL-C, HDL-C, TC, TG, FFA, ALT, AST, eGFR, HbA1c, CK, FBG, UACR, and HOMA-IR. If it is deemed necessary, effect of rosuvastatin on these parameters will be assessed using mixed model for longitudinal data. Due to the feasibility nature of this study, exploratory analysis may be performed. Continuous variables were described by mean and standard deviation, or by median and range; two-sided level of significance of 0.05 was applied to general comparison.

## 4. Conclusion

RNAFH study until now has been the first prospective, randomized controlled, open-label study initiated by investigator to explore the effect of rosuvastatin (10 mg/d) versus blank control for 52 weeks on IHCL evaluated by ^1^H-MRS in NAFLD patients with MS but without overt diabetes. The expected results of the study are that after 52 weeks of rosuvastatin treatment NAFLD patients could attain a lower IHCL and improved lipid parameters. Also, the side effects of this statin including insulin resistance level, hepatic dysfunction, muscle injury, renal dysfunction, and the incidence of overt diabetes will be within the acceptable range.
